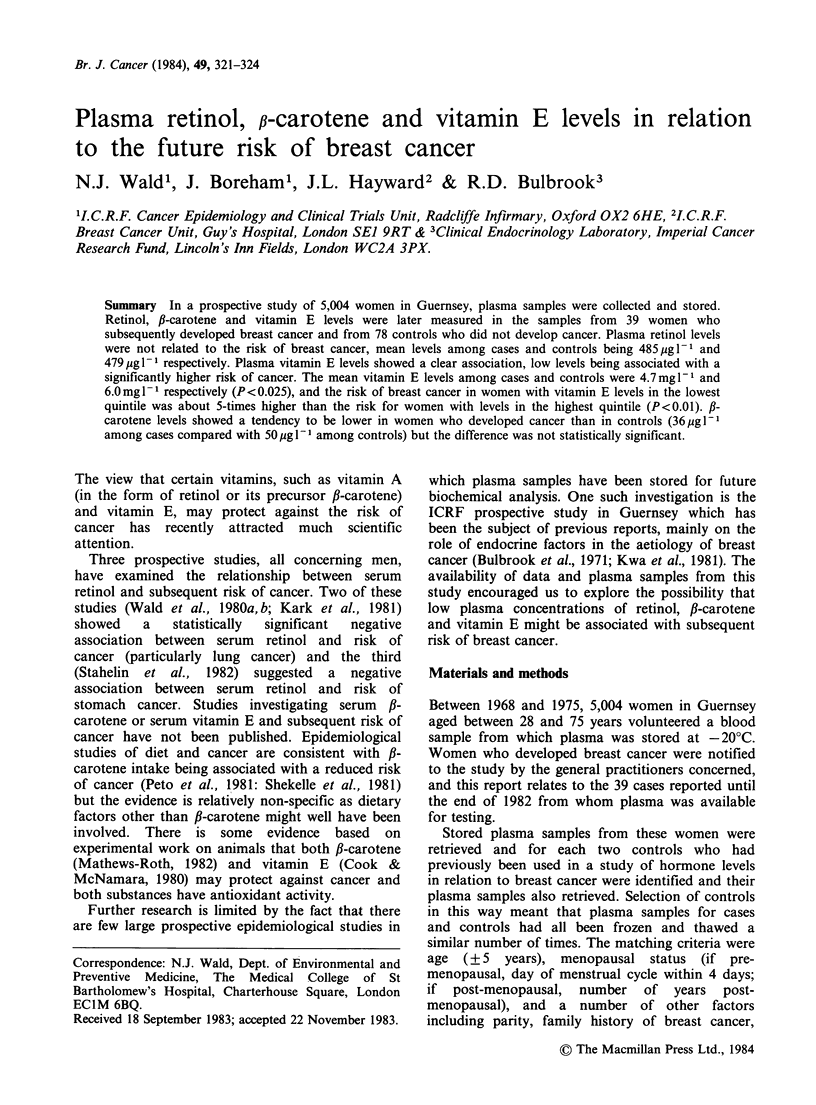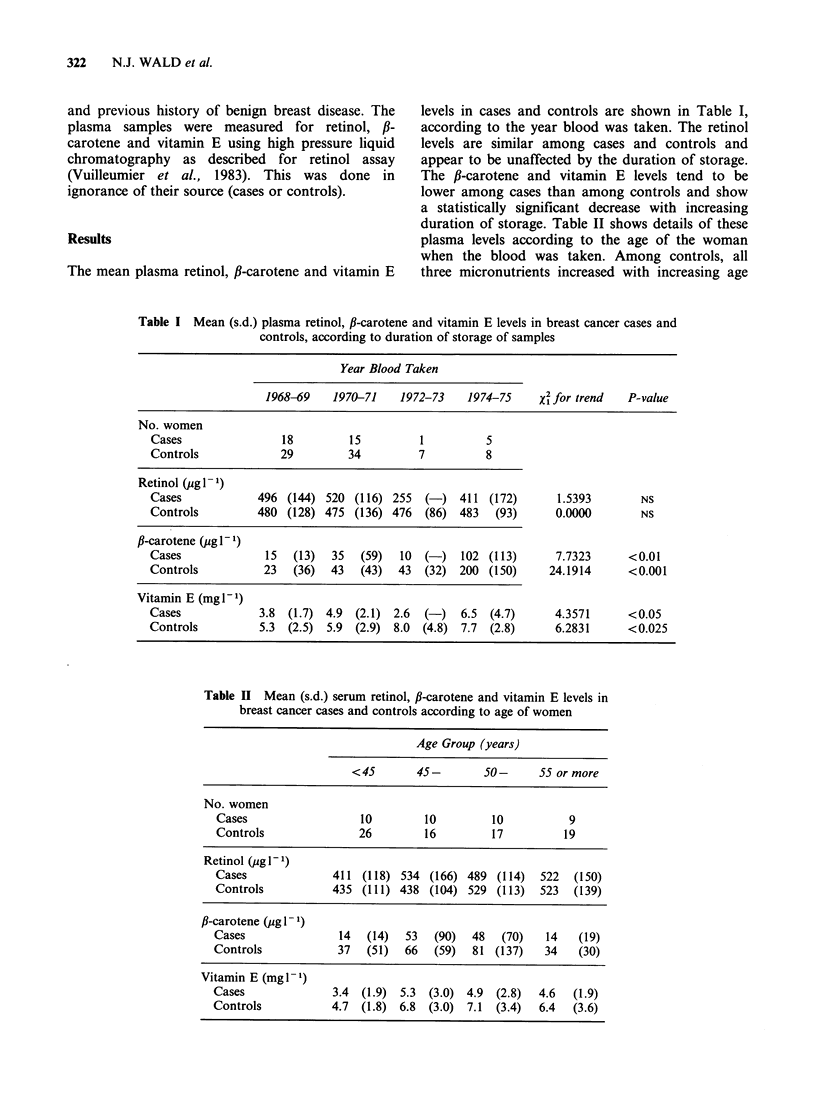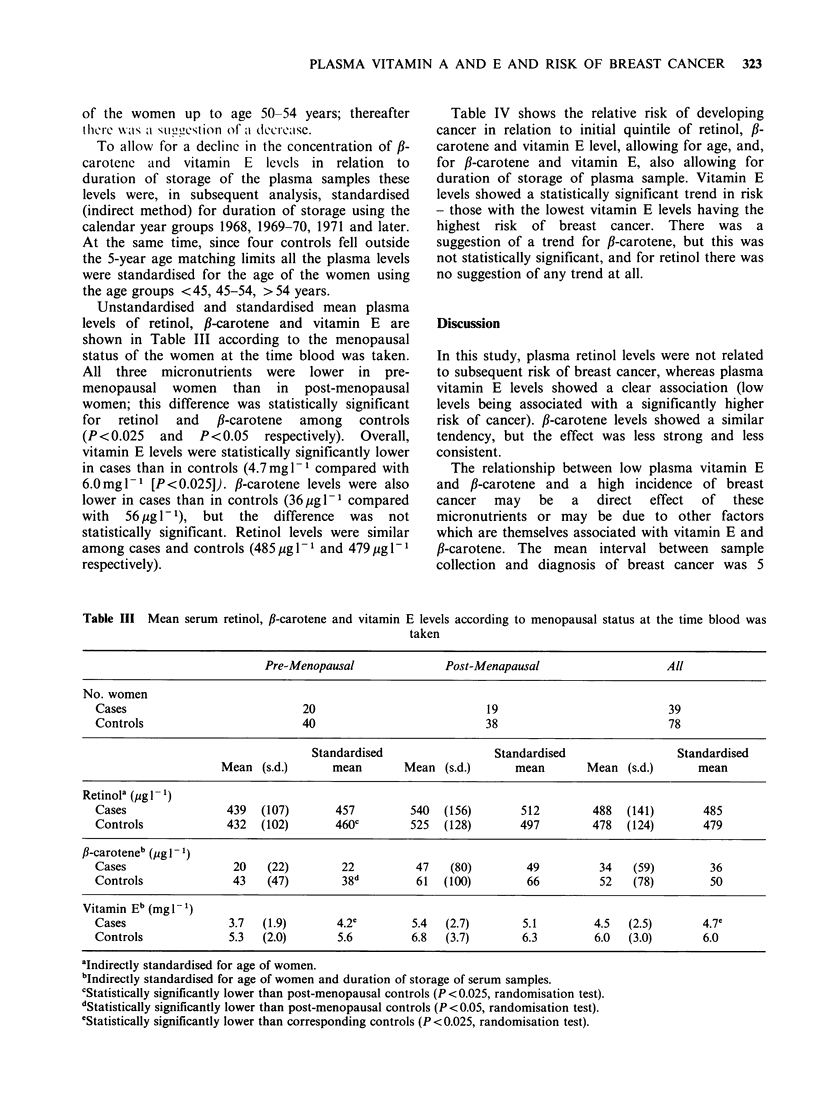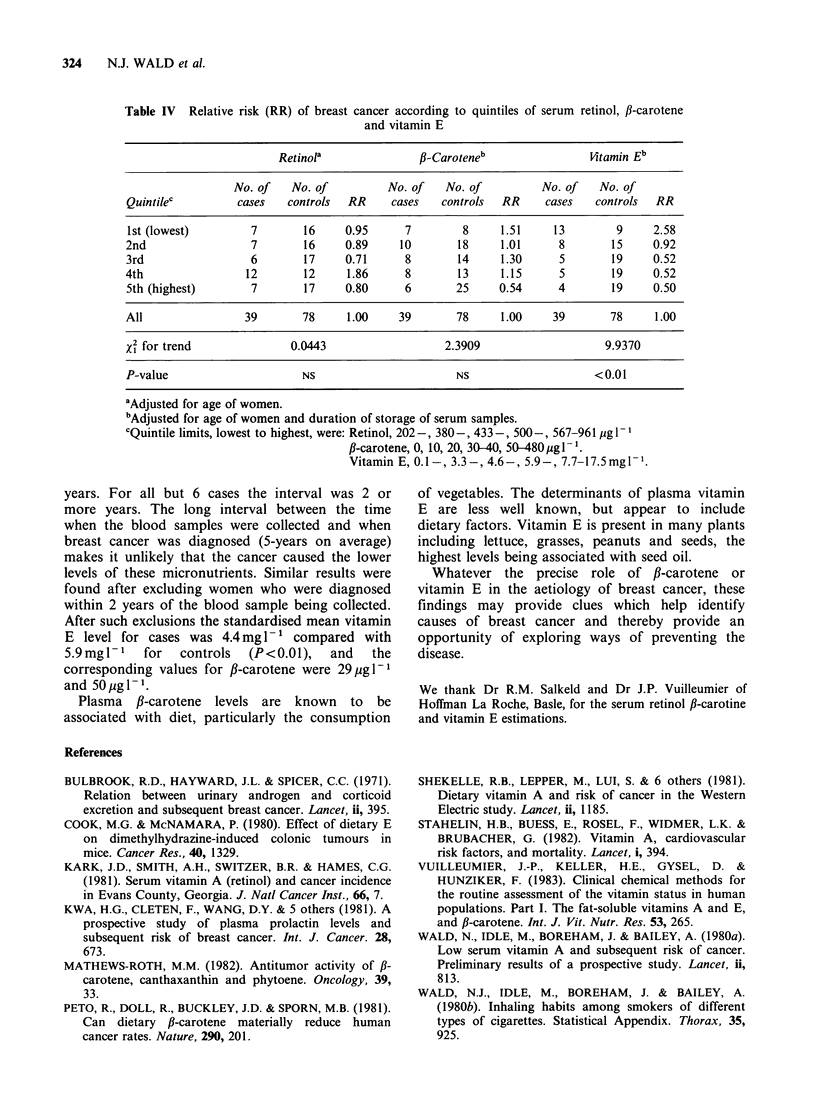# Plasma retinol, beta-carotene and vitamin E levels in relation to the future risk of breast cancer.

**DOI:** 10.1038/bjc.1984.50

**Published:** 1984-03

**Authors:** N. J. Wald, J. Boreham, J. L. Hayward, R. D. Bulbrook

## Abstract

In a prospective study of 5,004 women in Guernsey, plasma samples were collected and stored. Retinol, beta-carotene and vitamin E levels were later measured in the samples from 39 women who subsequently developed breast cancer and from 78 controls who did not develop cancer. Plasma retinol levels were not related to the risk of breast cancer, mean levels among cases and controls being 485 micrograms l-1 and 479 micrograms l-1 respectively. Plasma vitamin E levels showed a clear association, low levels being associated with a significantly higher risk of cancer. The mean vitamin E levels among cases and controls were 4.7 mg l-1 and 6.0 mg l-1 respectively (P less than 0.025), and the risk of breast cancer in women with vitamin E levels in the lowest quintile was about 5-times higher than the risk for women with levels in the highest quintile (P less than 0.01). beta-carotene levels showed a tendency to be lower in women who developed cancer than in controls (36 micrograms l-1 among cases compared with 50 micrograms l-1 among controls) but the difference was not statistically significant.


*Br. J. Cancer* (1984), **49**, 321–324

### Plasma retinol, β-carotene and vitamin E levels in relation to the future risk of breast cancer

N.J. Wald[1], J. Boreham[1], J.L. Hayward[2] & R.D. Bulbrook[3]

[1]*I.C.R.F. Cancer Epidemiology and Clinical Trials Unit, Radcliffe Infirmary, Oxford OX2 6HE,* [2]*I.C.R.F. Breast Cancer Unit, Guy's Hospital, London SE1 9RT &* [3]*Clinical Endocrinology Laboratory, Imperial Cancer Research Fund, Lincoln's Inn Fields, London WC2A 3PX.*

**Summary** In a prospective study of 5,004 women in Guernsey, plasma samples were collected and stored. Retinol, β-carotene and vitamin E levels were later measured in the samples from 39 women who subsequently developed breast cancer and from 78 controls who did not develop cancer. Plasma retinol levels were not related to the risk of breast cancer, mean levels among cases and controls being 485 $\mu g l^{-1}$ and 479 $\mu g l^{-1}$ respectively. Plasma vitamin E levels showed a clear association, low levels being associated with a significantly higher risk of cancer. The mean vitamin E levels among cases and controls were 4.7 $mg l^{-1}$ and 6.0 $mg l^{-1}$ respectively ($P<0.025$), and the risk of breast cancer in women with vitamin E levels in the lowest quintile was about 5-times higher than the risk for women with levels in the highest quintile ($P<0.01$). β-carotene levels showed a tendency to be lower in women who developed cancer than in controls (36 $\mu g l^{-1}$ among cases compared with 50 $\mu g l^{-1}$ among controls) but the difference was not statistically significant.

The view that certain vitamins, such as vitamin A (in the form of retinol or its precursor β-carotene) and vitamin E, may protect against the risk of cancer has recently attracted much scientific attention.

Three prospective studies, all concerning men, have examined the relationship between serum retinol and subsequent risk of cancer. Two of these studies (Wald *et al.*, 1980*a,b*; Kark *et al.*, 1981) showed a statistically significant negative association between serum retinol and risk of cancer (particularly lung cancer) and the third (Stahelin *et al.*, 1982) suggested a negative association between serum retinol and risk of stomach cancer. Studies investigating serum β-carotene or serum vitamin E and subsequent risk of cancer have not been published. Epidemiological studies of diet and cancer are consistent with β-carotene intake being associated with a reduced risk of cancer (Peto *et al.*, 1981: Shekelle *et al.*, 1981) but the evidence is relatively non-specific as dietary factors other than β-carotene might well have been involved. There is some evidence based on experimental work on animals that both β-carotene (Mathews-Roth, 1982) and vitamin E (Cook & McNamara, 1980) may protect against cancer and both substances have antioxidant activity.

Further research is limited by the fact that there are few large prospective epidemiological studies in which plasma samples have been stored for future biochemical analysis. One such investigation is the ICRF prospective study in Guernsey which has been the subject of previous reports, mainly on the role of endocrine factors in the aetiology of breast cancer (Bulbrook *et al.*, 1971; Kwa *et al.*, 1981). The availability of data and plasma samples from this study encouraged us to explore the possibility that low plasma concentrations of retinol, β-carotene and vitamin E might be associated with subsequent risk of breast cancer.

### Materials and methods

Between 1968 and 1975, 5,004 women in Guernsey aged between 28 and 75 years volunteered a blood sample from which plasma was stored at −20°C. Women who developed breast cancer were notified to the study by the general practitioners concerned, and this report relates to the 39 cases reported until the end of 1982 from whom plasma was available for testing.

Stored plasma samples from these women were retrieved and for each two controls who had previously been used in a study of hormone levels in relation to breast cancer were identified and their plasma samples also retrieved. Selection of controls in this way meant that plasma samples for cases and controls had all been frozen and thawed a similar number of times. The matching criteria were age (±5 years), menopausal status (if pre-menopausal, day of menstrual cycle within 4 days; if post-menopausal, number of years post-menopausal), and a number of other factors including parity, family history of breast cancer,

Correspondence: N.J. Wald, Dept. of Environmental and Preventive Medicine, The Medical College of St Bartholomew's Hospital, Charterhouse Square, London EC1M 6BQ.
Received 18 September 1983; accepted 22 November 1983.

© The Macmillan Press Ltd., 1984

and previous history of benign breast disease. The plasma samples were measured for retinol, β-carotene and vitamin E using high pressure liquid chromatography as described for retinol assay (Vuilleumier *et al.,* 1983). This was done in ignorance of their source (cases or controls).

### Results

The mean plasma retinol, β-carotene and vitamin E levels in cases and controls are shown in Table I, according to the year blood was taken. The retinol levels are similar among cases and controls and appear to be unaffected by the duration of storage. The β-carotene and vitamin E levels tend to be lower among cases than among controls and show a statistically significant decrease with increasing duration of storage. Table II shows details of these plasma levels according to the age of the woman when the blood was taken. Among controls, all three micronutrients increased with increasing age

**Table I** Mean (s.d.) plasma retinol, β-carotene and vitamin E levels in breast cancer cases and controls, according to duration of storage of samples

| | *Year Blood Taken* | | | | $\chi_1^2$ *for trend* | *P-value* |
|---|---|---|---|---|---|---|
| | *1968–69* | *1970–71* | *1972–73* | *1974–75* | | |
| No. women | | | | | | |
| Cases | 18 | 15 | 1 | 5 | | |
| Controls | 29 | 34 | 7 | 8 | | |
| Retinol ($\mu g\,l^{-1}$) | | | | | | |
| Cases | 496 (144) | 520 (116) | 255 (—) | 411 (172) | 1.5393 | NS |
| Controls | 480 (128) | 475 (136) | 476 (86) | 483 (93) | 0.0000 | NS |
| β-carotene ($\mu g\,l^{-1}$) | | | | | | |
| Cases | 15 (13) | 35 (59) | 10 (—) | 102 (113) | 7.7323 | <0.01 |
| Controls | 23 (36) | 43 (43) | 43 (32) | 200 (150) | 24.1914 | <0.001 |
| Vitamin E ($mg\,l^{-1}$) | | | | | | |
| Cases | 3.8 (1.7) | 4.9 (2.1) | 2.6 (—) | 6.5 (4.7) | 4.3571 | <0.05 |
| Controls | 5.3 (2.5) | 5.9 (2.9) | 8.0 (4.8) | 7.7 (2.8) | 6.2831 | <0.025 |

**Table II** Mean (s.d.) serum retinol, β-carotene and vitamin E levels in breast cancer cases and controls according to age of women

| | *Age Group (years)* | | | |
|---|---|---|---|---|
| | *<45* | *45–* | *50–* | *55 or more* |
| No. women | | | | |
| Cases | 10 | 10 | 10 | 9 |
| Controls | 26 | 16 | 17 | 19 |
| Retinol ($\mu g\,l^{-1}$) | | | | |
| Cases | 411 (118) | 534 (166) | 489 (114) | 522 (150) |
| Controls | 435 (111) | 438 (104) | 529 (113) | 523 (139) |
| β-carotene ($\mu g\,l^{-1}$) | | | | |
| Cases | 14 (14) | 53 (90) | 48 (70) | 14 (19) |
| Controls | 37 (51) | 66 (59) | 81 (137) | 34 (30) |
| Vitamin E ($mg\,l^{-1}$) | | | | |
| Cases | 3.4 (1.9) | 5.3 (3.0) | 4.9 (2.8) | 4.6 (1.9) |
| Controls | 4.7 (1.8) | 6.8 (3.0) | 7.1 (3.4) | 6.4 (3.6) |

of the women up to age 50–54 years; thereafter there was a suggestion of a decrease.

To allow for a decline in the concentration of β-carotene and vitamin E levels in relation to duration of storage of the plasma samples these levels were, in subsequent analysis, standardised (indirect method) for duration of storage using the calendar year groups 1968, 1969–70, 1971 and later. At the same time, since four controls fell outside the 5-year age matching limits all the plasma levels were standardised for the age of the women using the age groups <45, 45–54, >54 years.

Unstandardised and standardised mean plasma levels of retinol, β-carotene and vitamin E are shown in Table III according to the menopausal status of the women at the time blood was taken. All three micronutrients were lower in pre-menopausal women than in post-menopausal women; this difference was statistically significant for retinol and β-carotene among controls ($P<0.025$ and $P<0.05$ respectively). Overall, vitamin E levels were statistically significantly lower in cases than in controls ($4.7\,mg\,l^{-1}$ compared with $6.0\,mg\,l^{-1}$ [$P<0.025$]). β-carotene levels were also lower in cases than in controls ($36\,\mu g\,l^{-1}$ compared with $56\,\mu g\,l^{-1}$), but the difference was not statistically significant. Retinol levels were similar among cases and controls ($485\,\mu g\,l^{-1}$ and $479\,\mu g\,l^{-1}$ respectively).

Table IV shows the relative risk of developing cancer in relation to initial quintile of retinol, β-carotene and vitamin E level, allowing for age, and, for β-carotene and vitamin E, also allowing for duration of storage of plasma sample. Vitamin E levels showed a statistically significant trend in risk – those with the lowest vitamin E levels having the highest risk of breast cancer. There was a suggestion of a trend for β-carotene, but this was not statistically significant, and for retinol there was no suggestion of any trend at all.

### Discussion

In this study, plasma retinol levels were not related to subsequent risk of breast cancer, whereas plasma vitamin E levels showed a clear association (low levels being associated with a significantly higher risk of cancer). β-carotene levels showed a similar tendency, but the effect was less strong and less consistent.

The relationship between low plasma vitamin E and β-carotene and a high incidence of breast cancer may be a direct effect of these micronutrients or may be due to other factors which are themselves associated with vitamin E and β-carotene. The mean interval between sample collection and diagnosis of breast cancer was 5

**Table III** Mean serum retinol, β-carotene and vitamin E levels according to menopausal status at the time blood was taken

| | *Pre-Menopausal* | | | *Post-Menapausal* | | | *All* | | |
|---|---|---|---|---|---|---|---|---|---|
| No. women | | | | | | | | | |
| Cases | | 20 | | | 19 | | | 39 | |
| Controls | | 40 | | | 38 | | | 78 | |
| | Mean | (s.d.) | Standardised mean | Mean | (s.d.) | Standardised mean | Mean | (s.d.) | Standardised mean |
| Retinol[a] ($\mu g\,l^{-1}$) | | | | | | | | | |
| Cases | 439 | (107) | 457 | 540 | (156) | 512 | 488 | (141) | 485 |
| Controls | 432 | (102) | 460[c] | 525 | (128) | 497 | 478 | (124) | 479 |
| β-carotene[b] ($\mu g\,l^{-1}$) | | | | | | | | | |
| Cases | 20 | (22) | 22 | 47 | (80) | 49 | 34 | (59) | 36 |
| Controls | 43 | (47) | 38[d] | 61 | (100) | 66 | 52 | (78) | 50 |
| Vitamin E[b] ($mg\,l^{-1}$) | | | | | | | | | |
| Cases | 3.7 | (1.9) | 4.2[e] | 5.4 | (2.7) | 5.1 | 4.5 | (2.5) | 4.7[e] |
| Controls | 5.3 | (2.0) | 5.6 | 6.8 | (3.7) | 6.3 | 6.0 | (3.0) | 6.0 |

[a]Indirectly standardised for age of women.
[b]Indirectly standardised for age of women and duration of storage of serum samples.
[c]Statistically significantly lower than post-menopausal controls ($P<0.025$, randomisation test).
[d]Statistically significantly lower than post-menopausal controls ($P<0.05$, randomisation test).
[e]Statistically significantly lower than corresponding controls ($P<0.025$, randomisation test).

**Table IV** Relative risk (RR) of breast cancer according to quintiles of serum retinol, β-carotene and vitamin E

| | *Retinol*[a] | | | *β-Carotene*[b] | | | *Vitamin E*[b] | | |
|---|---|---|---|---|---|---|---|---|---|
| *Quintile*[c] | *No. of cases* | *No. of controls* | *RR* | *No. of cases* | *No. of controls* | *RR* | *No. of cases* | *No. of controls* | *RR* |
| 1st (lowest) | 7 | 16 | 0.95 | 7 | 8 | 1.51 | 13 | 9 | 2.58 |
| 2nd | 7 | 16 | 0.89 | 10 | 18 | 1.01 | 8 | 15 | 0.92 |
| 3rd | 6 | 17 | 0.71 | 8 | 14 | 1.30 | 5 | 19 | 0.52 |
| 4th | 12 | 12 | 1.86 | 8 | 13 | 1.15 | 5 | 19 | 0.52 |
| 5th (highest) | 7 | 17 | 0.80 | 6 | 25 | 0.54 | 4 | 19 | 0.50 |
| All | 39 | 78 | 1.00 | 39 | 78 | 1.00 | 39 | 78 | 1.00 |
| $\chi_1^2$ for trend | | 0.0443 | | | 2.3909 | | | 9.9370 | |
| *P*-value | | NS | | | NS | | | <0.01 | |

[a]Adjusted for age of women.
[b]Adjusted for age of women and duration of storage of serum samples.
[c]Quintile limits, lowest to highest, were: Retinol, 202–, 380–, 433–, 500–, 567–961 $\mu g\,l^{-1}$
β-carotene, 0, 10, 20, 30–40, 50–480 $\mu g\,l^{-1}$.
Vitamin E, 0.1–, 3.3–, 4.6–, 5.9–, 7.7–17.5 $mg\,l^{-1}$.

years. For all but 6 cases the interval was 2 or more years. The long interval between the time when the blood samples were collected and when breast cancer was diagnosed (5-years on average) makes it unlikely that the cancer caused the lower levels of these micronutrients. Similar results were found after excluding women who were diagnosed within 2 years of the blood sample being collected. After such exclusions the standardised mean vitamin E level for cases was 4.4 $mg\,l^{-1}$ compared with 5.9 $mg\,l^{-1}$ for controls ($P<0.01$), and the corresponding values for β-carotene were 29 $\mu g\,l^{-1}$ and 50 $\mu g\,l^{-1}$.

Plasma β-carotene levels are known to be associated with diet, particularly the consumption of vegetables. The determinants of plasma vitamin E are less well known, but appear to include dietary factors. Vitamin E is present in many plants including lettuce, grasses, peanuts and seeds, the highest levels being associated with seed oil.

Whatever the precise role of β-carotene or vitamin E in the aetiology of breast cancer, these findings may provide clues which help identify causes of breast cancer and thereby provide an opportunity of exploring ways of preventing the disease.

We thank Dr R.M. Salkeld and Dr J.P. Vuilleumier of Hoffman La Roche, Basle, for the serum retinol β-carotine and vitamin E estimations.

### References

BULBROOK, R.D., HAYWARD, J.L. & SPICER, C.C. (1971). Relation between urinary androgen and corticoid excretion and subsequent breast cancer. *Lancet*, **ii**, 395.

COOK, M.G. & McNAMARA, P. (1980). Effect of dietary E on dimethylhydrazine-induced colonic tumours in mice. *Cancer Res.*, **40**, 1329.

KARK, J.D., SMITH, A.H., SWITZER, B.R. & HAMES, C.G. (1981). Serum vitamin A (retinol) and cancer incidence in Evans County, Georgia. *J. Natl Cancer Inst.*, **66**, 7.

KWA, H.G., CLETEN, F., WANG, D.Y. & 5 others (1981). A prospective study of plasma prolactin levels and subsequent risk of breast cancer. *Int. J. Cancer*. **28**, 673.

MATHEWS-ROTH, M.M. (1982). Antitumor activity of β-carotene, canthaxanthin and phytoene. *Oncology*, **39**, 33.

PETO, R., DOLL, R., BUCKLEY, J.D. & SPORN, M.B. (1981). Can dietary β-carotene materially reduce human cancer rates. *Nature*, **290**, 201.

SHEKELLE, R.B., LEPPER, M., LUI, S. & 6 others (1981). Dietary vitamin A and risk of cancer in the Western Electric study. *Lancet*, **ii**, 1185.

STAHELIN, H.B., BUESS, E., ROSEL, F., WIDMER, L.K. & BRUBACHER, G. (1982). Vitamin A, cardiovascular risk factors, and mortality. *Lancet*, **i**, 394.

VUILLEUMIER, J.-P., KELLER, H.E., GYSEL, D. & HUNZIKER, F. (1983). Clinical chemical methods for the routine assessment of the vitamin status in human populations. Part I. The fat-soluble vitamins A and E, and β-carotene. *Int. J. Vit. Nutr. Res.* **53**, 265.

WALD, N., IDLE, M., BOREHAM, J. & BAILEY, A. (1980*a*). Low serum vitamin A and subsequent risk of cancer. Preliminary results of a prospective study. *Lancet*, **ii**, 813.

WALD, N.J., IDLE, M., BOREHAM, J. & BAILEY, A. (1980*b*). Inhaling habits among smokers of different types of cigarettes. Statistical Appendix. *Thorax*, **35**, 925.